# QuEChERS-超高效液相色谱-串联质谱法快速测定水产品中5种卤代苯醌

**DOI:** 10.3724/SP.J.1123.2022.09020

**Published:** 2023-06-08

**Authors:** Xuezhi TONG, Dongyang CHEN, Jiali FENG, Xiang FAN, Hao ZHANG, Shengyuan YANG

**Affiliations:** 1.南华大学公共卫生学院, 湖南 衡阳 421001; 1. Department of Public Health Laboratory Sciences, School of Public Health, Hengyang Medical School, University of South China, Hengyang 421001, China; 2.湖南省疾病预防控制中心, 湖南 长沙 410005; 2. Hunan Provincial Center for Disease Control and Prevention, Changsha 410005, China

**Keywords:** QuEChERS, 超高效液相色谱-串联质谱法, 卤代苯醌, 水产品, QuEChERS, ultra performance liquid chromatography-tandem mass spectrometry (UPLC-MS/MS), halobenzoquinones (HBQs), aquatic products

## Abstract

随着消毒剂在环境中的广泛使用,水体中卤代苯醌(HBQs)存在的风险逐渐升高,建立水产品中HBQs的检测方法具有重要的现实意义。本研究建立了基于QuEChERS-超高效液相色谱-串联质谱(UPLC-MS/MS)快速测定水产品中5种HBQs含量的方法。选择10%甲醇乙腈溶液(含0.1%甲酸)为提取溶剂,加入氯化钠和无水硫酸镁脱水离心,采用50 mg *N*-丙基乙二胺(PSA)、30 mg石墨化炭黑(GCB)和30 mg中性氧化铝(Al_2_O_3_)组合吸附剂对上清液进行吸附净化,净化液经氮气吹至近干,乙腈复溶后上机测定。待测物以0.25%甲酸乙腈溶液和0.25%甲酸水溶液为流动相,通过Waters ACQUITY UPLC BEH C_18_色谱柱(100 mm×2.1 mm, 1.7 μm)分离,在电喷雾负离子(ESI^-^)、多反应监测(MRM)模式下进行测定,采用基质匹配标准曲线定量。5种HBQs在6 min内可达到较好的色谱分离,同时通过空白基质加标工作曲线评价基质效应,其中2,5-二氯-1,4-苯醌(2,5-DCBQ)存在基质增强效应,其余HBQs为基质抑制效应,特别是四氯苯醌(TCBQ)呈现强基质抑制效应。在优化的条件下,5种HBQs在1.0~50.0 μg/L范围内具有良好的线性关系,相关系数(*r*)≥0.9992,方法检出限为0.15~0.8 μg/kg;在低、中、高3个加标水平下,5种HBQs的加标回收率为85.9%~116.5%,相对标准偏差(RSD)为1.4%~8.2%。该方法可实现HBQs在水产品中的快速富集与净化,具有灵敏度低、操作简便、重复性好等优势,可为水产品中痕量HBQs的大规模监测用提供技术支持。

## Abstract (en)

Halobenzoquinones (HBQs), which are emerging chlorinated disinfection byproducts (DBPs), have attracted increasing attention because they are frequently detected in treated tap water, entrainment water, etc. These compounds are mainly generated during the water treatment process using chlorine, chloramine, and chlorine dioxide as disinfectants, and display more toxic effects than regulated DBPs, such as trihalomethane and haloacetic acid. HBQs have been recognized as potential bladder carcinogens and are harmful to the nervous system. Additionally, they can exert genotoxic effects and cause oxidative damage to DNA and proteins. The risk of HBQs in aquatic products is expected to rise because the disinfection of public facilities has significantly increased in recent years. Therefore, developing a sensitive and accurate analytical method to detect HBQs in aquatic products is of great importance. Several analytical methods, including gas chromatography, gas chromatography-mass spectrometry, electrochemical methods, liquid chromatography, and liquid chromatography-tandem mass spectrometry, can be used to identify and quantify HBQs in water. However, to the best of our knowledge, no reports on the determination of HBQ levels in aquatic products are yet available. Further, pretreatment is essential for HBQ determination because of the complex matrix effects of aquatic products. Herein, a sensitive and accurate method based on the QuEChERS technique coupled with ultra performance liquid chromatography-tandem mass spectrometry (UPLC-MS/MS) was developed for the simultaneous determination of five HBQs in aquatic products. For the QuEChERS procedure, the pretreatment conditions, such as the extraction solvent and adsorbent species, were systematically optimized. The sample was extracted with 10 mL of 10% methanol acetonitrile solution (containing 0.1% formic acid), dehydrated, and centrifuged with sodium chloride and anhydrous magnesium sulfate. The supernatant was purified using a QuEChERS packing material consisting of 50 mg *N*-propylethylenediamine (PSA), 30 mg of graphitized carbon black (GCB), and 30 mg of neutral alumina (Al_2_O_3_), dried with nitrogen, and concentrated. The five HBQs were separated on a Waters ACQUITY UPLC BEH C_18_ column (100 mm×2.1 mm, 1.7 μm) using 0.25% acetonitrile formate solution and 0.25% formic acid aqueous solution as the mobile phase under a gradient elution program and then detected using UPLC-MS/MS with negative electrospray ionization (ESI^-^) under multiple reaction monitoring (MRM) mode. Quantitative analysis was performed using a matrix-matched external standard method. The five HBQs achieved rapid separation within 6 min, indicating that the proposed method has a much shorter separation time compared with previous studies. The matrix effect was evaluated by establishing a matrix-matched calibration curve. The results showed that 2,5-dichloro-1,4-benzoquinone (2,5-DCBQ) presented a matrix-enhancing effect, whereas the other HBQs displayed matrix-inhibiting effects. In particular, tetrachlorobenzoquinone (TCBQ) exhibited strong inhibitory effects. Under the optimized experimental conditions, the five HBQs demonstrated good linear relationships in the range of 1.0-50.0 μg/L, with correlation coefficients (*r*)≥0.9992. The detection limits of the method were 0.15-0.8 μg/kg, and the recoveries of the target compounds were 85.9%-116.5%. The relative standard deviations were 1.4%-8.2%, which indicates good reproducibility. The proposed method was successfully applied to actual sample detection, and 2,6-dichloro-3-methyl-1,4-benzoquinone (2,6-DCMBQ) was detected in grass carp. The proposed method is convenient, sensitive, accurate, and suitable for the simultaneous determination of five HBQs in aquatic products. Moreover, the developed method provides a reliable reference for the routine monitoring of trace HBQs in food samples.

卤代苯醌(halobenzoquinones, HBQs)是近年来被广泛关注的一类新型消毒副产物^[1]^,主要是由液氯、氯胺、二氧化氯等含氯消毒剂对水进行处理后产生的^[2]^。前期通过对饮用水、泳池水等水体进行调查,发现在饮用水、泳池水等经氯消毒处理过的水体中普遍检出HBQs^[3,4]^。HBQs在饮用水中含量虽低,但毒理学研究表明其相对于已受控的消毒副产物如三卤甲烷、卤乙酸等具有更强的细胞毒性和遗传毒性,是膀胱癌的致癌物^[5]^。此外,HBQs还具有神经毒性和生殖发育毒性^[6,7]^,易诱导哺乳动物染色体损伤^[8]^。近年来随着对环境、排水设施和污水处理等公共设施消杀行为的大幅增加,含氯消毒剂排放至地表水从而产生HBQs的风险增大,从而导致水生生物表面和体内存在HBQs的可能性也随之增大。尽管目前尚无关于HBQs在水产品中代谢的研究报道,但研究表明HBQs在酸性条件下稳定,碱性条件下则会水解生成带有羟基自由基的卤代苯醌(OH-HBQ)^[9,10]^。新鲜水产品如鲫鱼、草鱼等为酸性食品,且其在冷藏初期经糖原酵解反应生成的乳酸、腺嘌呤核苷三磷酸(ATP)和磷酸肌酸等物质可分解为磷酸等酸性物质,导致体内的pH进一步降低^[11,12]^,在此条件下,附着在水产品表面和存在于水产品体内的HBQs处于稳定状态。此外,当前HBQs的生理毒性研究均是以其原型对水产品如斑马鱼或体外细胞等生物模型的危害展开的^[13,14]^。因此,建立水产品中HBQs的测定方法,对有效评估人体经水产品摄入HBQs的暴露风险具有重要的实际应用价值。

目前HBQs的测定方法主要有气相色谱法^[15]^、气相色谱-质谱法^[16]^、纳米传感器法^[17]^、电化学法^[18]^、液相色谱法^[19]^和液相色谱-串联质谱法^[20]^等,其中气相色谱和气相色谱-质谱法适用于易挥发、热稳定性好的HBQs,但大部分HBQs具有热不稳定的特性;液相色谱-串联质谱法灵敏度高,定性能力强,是分析HBQs的首选方法。此外,水产品基质复杂,含有丰富的蛋白质和脂肪,一般需对样品进行前处理。常见的前处理方法包括加速溶剂萃取^[21]^、分散固相萃取^[22]^、固相萃取^[23]^、固相微萃取^[24]^等,但传统的样品前处理技术存在操作繁琐、环境不友好等缺点,QuEChERS方法操作简单,能有效去除有机酸、脂肪酸、碳水化合物等,易实现高通量处理,适合大批量样品的监测。

因此,本研究基于QuEChERS前处理技术,以水产品中HBQs为研究对象,通过优化前处理条件,建立了一种可以快速同时测定水产品中5种HBQs的分析方法,并对方法性能进行了系统考察。该方法将为研究HBQs在生物体内的形成途径、含量和控制提供强有力的技术支撑,对保护公共卫生安全与消费者身体健康具有重要的现实意义。

## 1 实验部分

### 1.1 试剂与仪器

标准品:四氯苯醌(TCBQ, FS1620696, 纯度98.6%)、2,5-二氯-1,4-苯醌(2,5-DCBQ, FS1620697, 纯度99.1%)、2,6-二氯-1,4-苯醌(2,6-DCBQ, FS1620695, 纯度99.6%)、2,6-二溴-1,4-苯醌(2,6-DBBQ, FS1621996, 纯度98.1%)和2,6-二氯-3-甲基-1,4-苯醌(2,6-DCMBQ, FS1621995, 纯度99.2%)均购买于中国天津Medjen公司。

氯化钠、无水硫酸镁、中性氧化铝(均为优级纯)购买于国药集团化学试剂有限公司,*N*-丙基乙二胺(PSA)购自美国安捷伦公司,石墨化炭黑(GCB)购自上海安谱实验科技股份有限公司;乙腈、甲醇、二氯甲烷和乙酸乙酯(均为色谱纯)均购自美国Merck公司; Waters XEVOTQ-S三重四极杆质谱仪(美国Waters公司);TI-H-15超声波清洗仪(德国Elma公司); VORTEX-GENIE 2涡旋混匀器(海门市其林贝尔仪器制造有限公司); TGL16高速冷冻离心机(长沙英泰仪器有限公司);实验用水为经Milli-Q处理的超纯水(美国Millipore公司)。

### 1.2 标准溶液的配制

分别称取10 mg TCBQ、2,5-DCBQ、2,6-DCBQ、2,6-DBBQ、2,6-DCMBQ标准品,用乙腈溶解、定容至10 mL,配制成质量浓度为1.0 mg/mL的标准储备液。移取0.10 mL标准储备液至10 mL容量瓶中,用0.25%甲酸乙腈溶液稀释为10.0 mg/L的标准中间液,这些标准溶液均在-18 ℃下避光保存。

### 1.3 样品前处理

取水产品样品可食用部分进行匀浆,准确称取1.0 g均匀样品于15 mL离心管中,用10 mL 10%甲醇乙腈溶液(含0.1%甲酸)超声提取10 min,加入0.5 g NaCl和0.5 g无水MgSO_4_,充分涡旋后震荡5 min;以7500 r/min离心5 min,取上清液4.0 mL,加入50 mg PSA、30 mg GCB和30 mg中性Al_2_O_3_,涡旋混匀后以7500 r/min离心5 min,取上清液2 mL,氮吹至近干,加入乙腈复溶至1.0 mL,再经0.22 μm尼龙膜过滤,待UPLC-MS/MS测定。

### 1.4 分析条件

#### 1.4.1 色谱条件

色谱柱:Waters ACQUITY UPLC BEH C_18_柱(100 mm×2.1 mm, 1.7 μm);柱温30 ℃;进样量5 μL。流动相A为0.25%甲酸水溶液,B为0.25%甲酸乙腈溶液,流速0.3 mL/min。梯度洗脱程序:0~2.0 min, 80.0%A~60.0%A; 2.0~4.0 min, 60.0%A~35.0%A; 4.0~5.0 min, 35.0%A; 5.0~5.1 min, 35.0%A~80%A; 5.1~6.0 min, 80%A。

#### 1.4.2 质谱条件

电喷雾离子源(ESI),负离子扫描模式,多反应监测(MRM)模式下进行检测;毛细管电压0.50 kV;脱溶剂气流量1000 L/h;脱溶剂温度550 ℃;锥孔气流量150 L/h; 5种HBQs的MRM模式质谱分析参数及保留时间见表1。

**表1:** MRM模式下5种HBQs的质谱参数

Compound	Parent ion (m/z)	Daughter ions (m/z)	CV/V	CE/eV
2,5-Dichloro-1,4-benzoquinone (2,5-DCBQ)	177	113^*^/35	27	10/16
2,6-Dichloro-1,4-benzoquinone (2,6-DCBQ)	177	113^*^/141	35	17/10
2,6-Dibromo-1,4-benzoquinone (2,6-DBBQ)	267	81^*^/79	40	22
2,6-Dichloro-3-methyl-1,4-benzoquinone (2,6-DCMBQ)	191	127^*^/155	30	15/9
Tetrachlorobenzoquinone (TCBQ)	246	209^*^/182	42	14/13

CV: cone voltage; CE: collision energy; *quantitative ion.

## 2 结果与讨论

### 2.1 色谱条件优化

HBQs在中性和碱性环境下易发生水解,在流动相中添加甲酸可以有效提高HBQs的稳定性,促进其在电喷雾过程中的电离,增强响应。但Wang等^[25]^认为流动相中添加弱酸会抑制电喷雾负离子电离的信号。因此分别比较了水-甲醇、水-乙腈、0.1%甲酸水溶液-乙腈、0.25%甲酸水溶液-乙腈、0.25%甲酸水溶液-0.25%甲酸乙腈作为流动相时目标物的响应情况。实验发现,乙腈作为有机相时分离度优于甲醇,流动相加酸后响应值增强,加入0.25%甲酸时的响应值明显高于0.1%甲酸。因此确定最优流动相条件为0.25%甲酸水溶液-0.25%甲酸乙腈,在该条件下2,5-DCBQ与2,6-DCBQ可实现基线分离,5种待测物在6 min内可实现有效色谱分离,较先前的研究显著缩短了色谱分离时间^[3]^。5种HBQs的总离子流色谱图见图1。

**图1:**
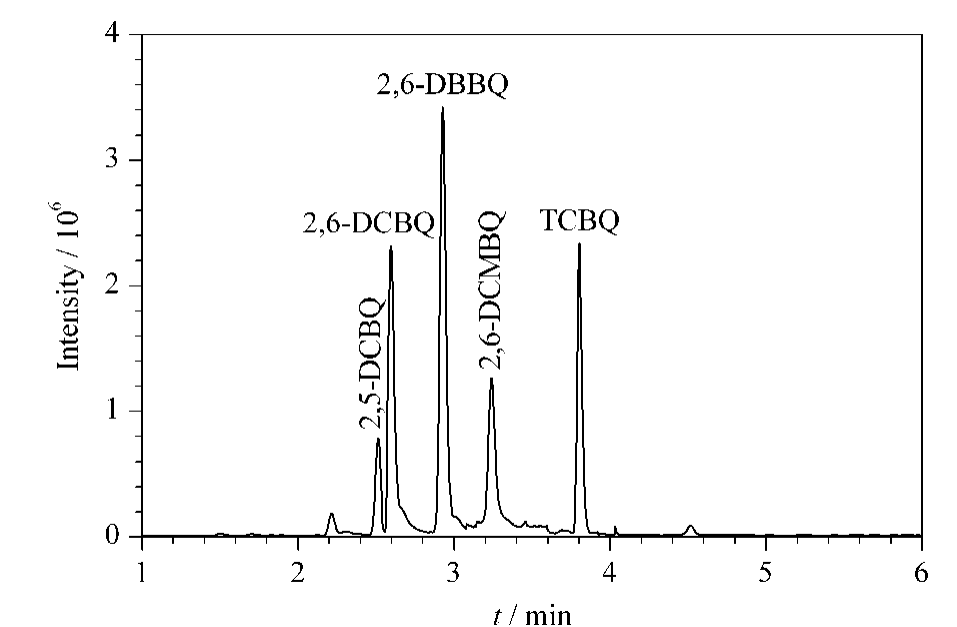
5种HBQs混合标准溶液的总离子流色谱图

### 2.2 前处理条件的优化

#### 2.2.1 提取溶剂的选择

测定水产品中HBQs的主要问题是样品存在脂质干扰,由于乙腈极性大,油脂等非极性杂质不易被提取出来,且对蛋白质有一定的沉淀效果。因此分别考察了乙腈、10%二氯甲烷乙腈溶液、10%甲醇乙腈溶液、10%乙酸乙酯乙腈溶液作为提取溶剂的提取效果。结果如图2所示,10%甲醇乙腈溶液的提取效果最佳,5种HBQs的回收率为81.2%~119.3%,尤其对TCBQ的回收效果最佳,回收率可达83.6%,且提取的样品杂质较加入二氯甲烷和乙酸乙酯时少。HBQs在碱性和中性环境下易发生水解,在酸性条件下稳定性可得到改善^[26]^。因此采用加酸的方式来稳定体系中的HBQs,进一步考察提取溶剂中添加不同体积分数甲酸(0、0.1%、0.25%、0.5%)的10%甲醇乙腈溶液对目标物的提取效果,结果如图3所示。添加甲酸后,HBQs的回收率得到改善(88.6%~106.4%), TCBQ的回收率由83.6%上升至100.4%,但待测物的提取效果与酸浓度关系不大,随着甲酸体积分数增加,2,6-DBBQ、2,5-DCBQ、2,6-DCBQ的回收率反而出现回落。因此,确定提取溶剂中甲酸的体积分数为0.1%。

**图2:**
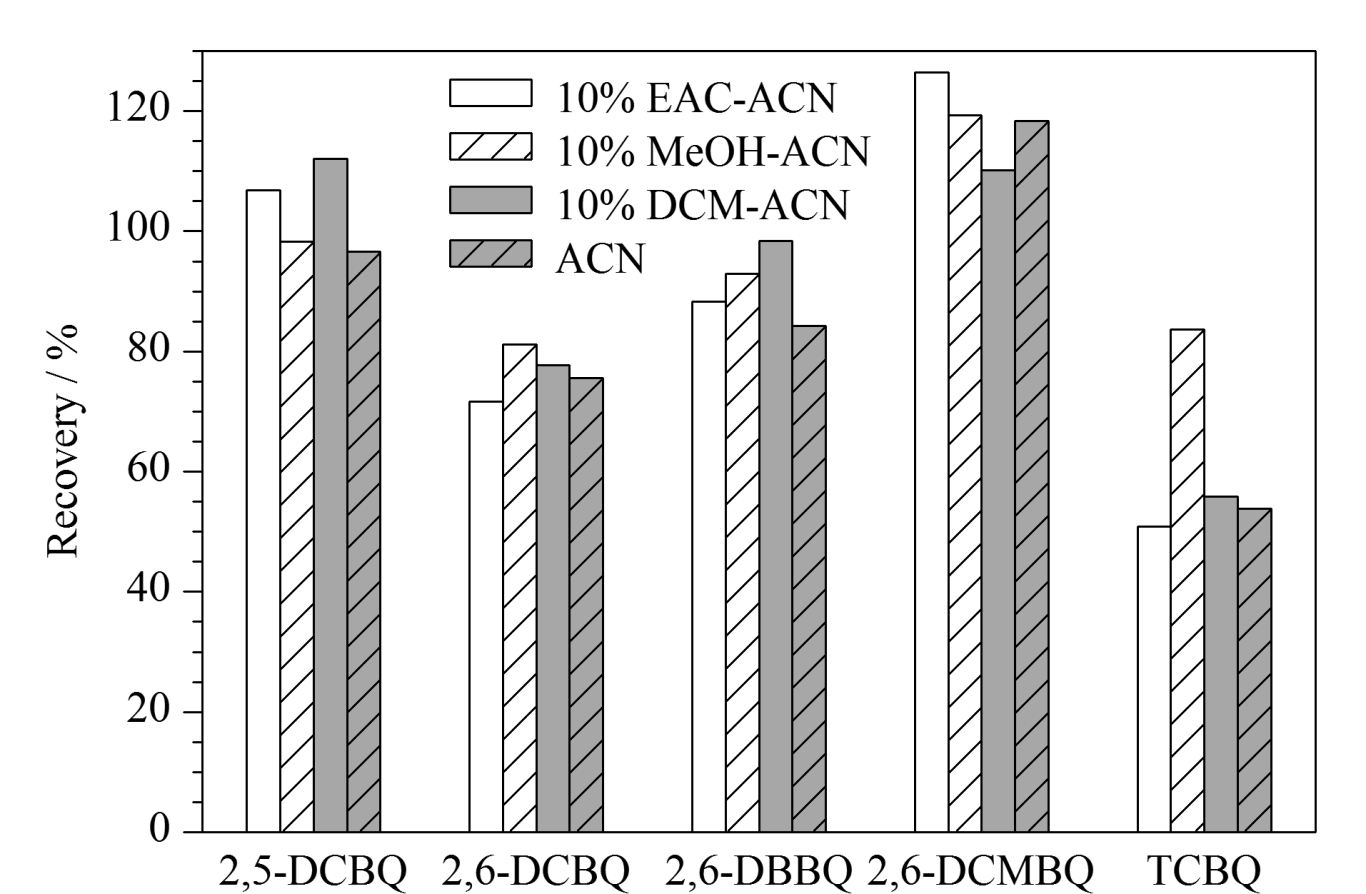
不同提取溶剂对5种HBQs回收率的影响

**图3:**
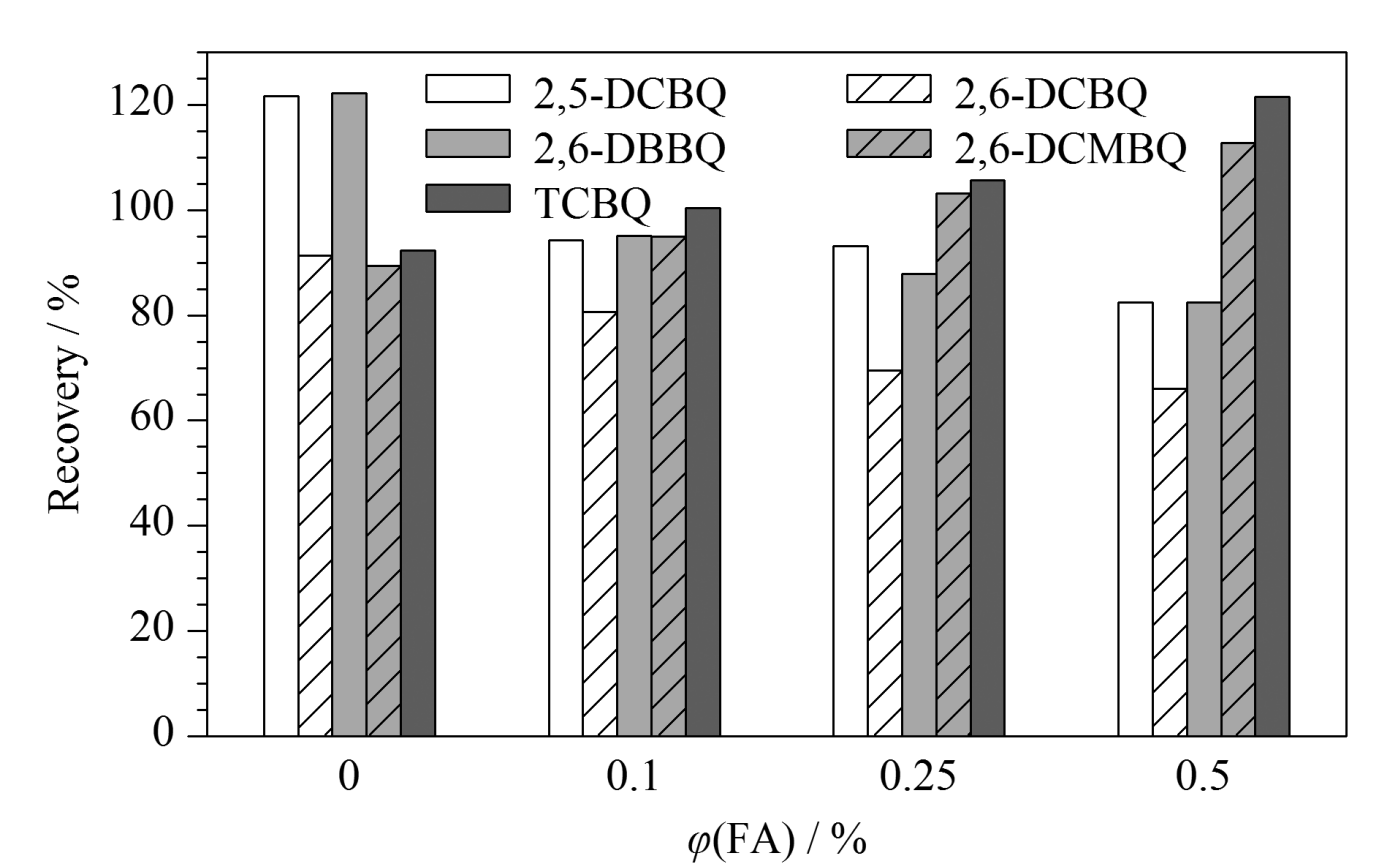
提取溶剂中不同体积分数的甲酸对5种HBQs回收率的影响

#### 2.2.2 吸附剂的优化

在QuEChERS方法中,PSA能去除样品中的多糖、脂肪酸以及其他有机酸等杂质,中性Al_2_O_3_吸附剂能去除蛋白质,GCB能去除部分色素、固醇和具有平面结构的基质干扰物。基于以上3种常用的吸附剂,对其单独及组合使用(PSA(100 mg)、PSA+GCB(50 mg+50 mg)、PSA+Al_2_O_3_(50 mg+50 mg)、PSA+GCB+Al_2_O_3_(50 mg+30 mg+30 mg))的净化效果进行了考察。

结果如图4,加入吸附剂GCB后,TCBQ的回收率增大,2,6-DCMBQ的基质抑制效应减弱,而在加入中性Al_2_O_3_后,基质抑制效应未得到明显改善,推测2,6-DCMBQ的检测主要受固醇等物质的干扰;而2,6-DBBQ和2,6-DCBQ在吸附剂为PSA+GCB+Al_2_O_3_的组合下获得了最佳回收率,分别为110.5%和90.7%。因此选用PSA+GCB+Al_2_O_3_的组合吸附剂进行后续实验。

**图4:**
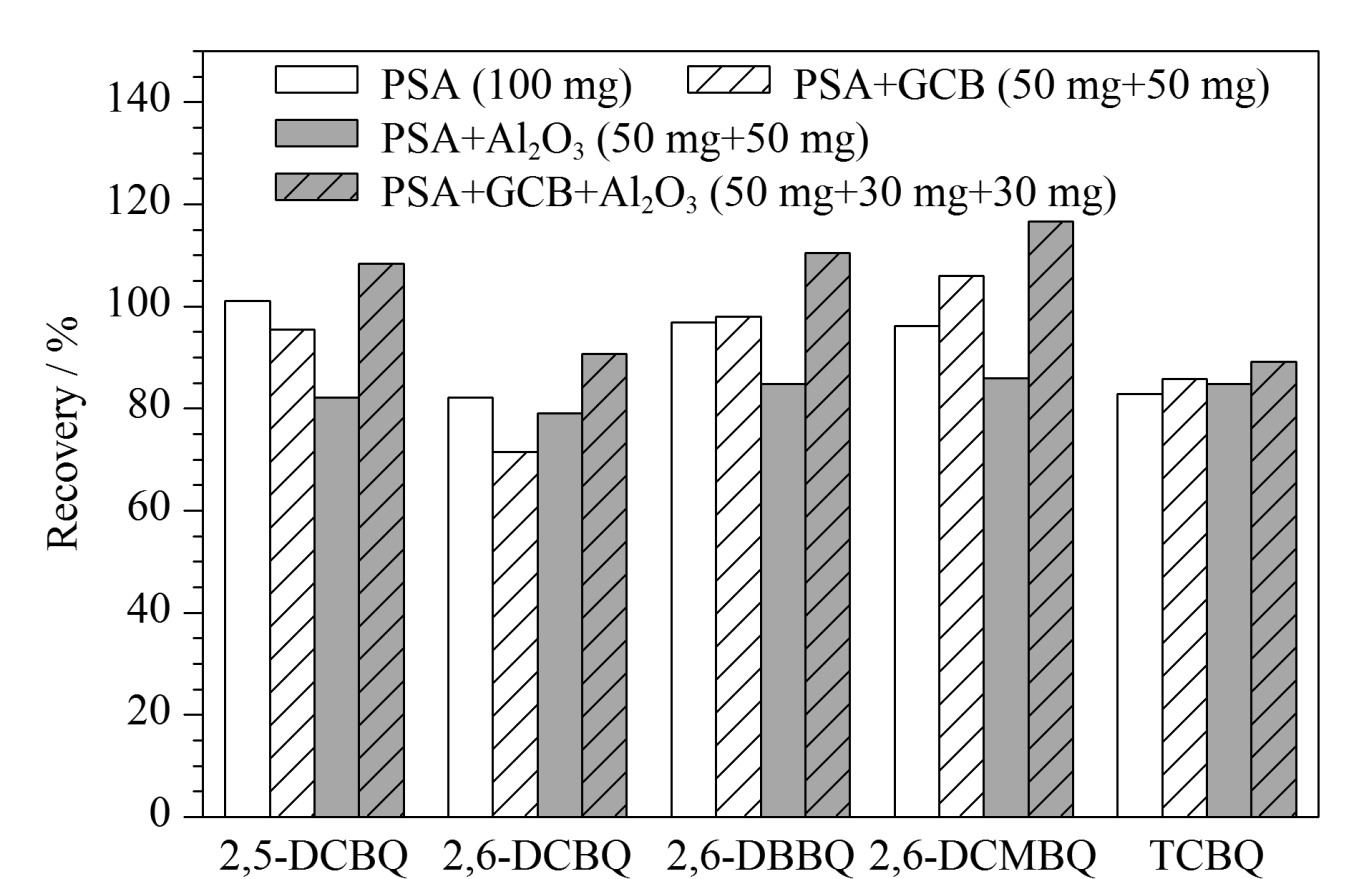
不同吸附剂组合对5种HBQs回收率的影响

### 2.3 基质效应

通过比较鲈鱼和金鲳鱼的空白基质加标曲线与空白溶剂加标曲线来评价基质效应(ME)。计算公式为ME=(基质匹配标准曲线斜率/溶剂标准曲线斜率-1)×100%。当|ME|<20%时为弱基质效应;当20%≤|ME|≤50%|时为中等基质效应;当|ME|>50%时为强基质效应。ME为负值表示存在基质抑制效应,正值表示存在基质增强效应^[27]^。结果见表2,鲈鱼和金鲳鱼基质中HBQs多为基质抑制效应,其中TCBQ的基质效应分别为-80.8%和-78.2%,具有明显基质抑制效应;2,6-DBBQ、2,6-DCMBQ、2,6-DCBQ的基质效应为-28.0%~-12.6%,影响较小,而2,5-DCBQ存在基质增强效应。本实验采用空白基质提取液配制混合标准溶液进行校正,经校正后可有效消除基质效应带来的影响,提高检测结果的准确性。

**表2:** 5种HBQs在鲈鱼和金鲳鱼中的基质效应

Compound	Matrix effects/%	
	Weever	Trachinotus ovatus
2,5-DCBQ	19.1	20.1
2,6-DCBQ	-28.0	-21.2
2,6-DBBQ	-12.6	-18.7
2,6-DCMBQ	-18.3	-12.7
TCBQ	-80.8	-78.2

### 2.4 方法学评价

#### 2.4.1 线性范围与方法检出限

将1.0 mg/L混合标准溶液用鲈鱼空白基质样品溶液稀释成质量浓度为1.0、5.0、10.0、20.0、50.0 μg/L的标准工作溶液,以目标物峰面积为纵坐标(*y*),对应的质量浓度为横坐标(*x*, μg/L)绘制标准工作曲线并计算回归方程,分别以3倍和10倍信噪比(*S/N*)确定仪器检出限和仪器定量限,按照方法处理步骤,计算方法检出限(LOD)和方法定量限(LOQ)。实验结果表明,5种HBQs在1.0~50.0 μg/L范围内均具有良好的线性关系,相关系数(*r*)≥0.9992。方法检出限和方法定量限分别为0.15~0.8 μg/kg和0.45~2.5 μg/kg,相关实验数据见表3。

**表3:** 5种HBQs的线性范围、线性方程、相关系数、方法检出限和方法定量限

Compound	Linear range/(μg/L)	Linear equation	r	LOD/(μg/kg)	LOQ/(μg/kg)
2,5-DCBQ	1.0-50.0	y=14781x-4180.9	0.9999	0.15	0.45
2,6-DCBQ	1.0-50.0	y=8169.9x-7139.4	0.9997	0.3	1.0
2,6-DBBQ	1.0-50.0	y=7145.2x-1756.7	0.9999	0.3	1.0
2,6-DCMBQ	1.0-50.0	y=2869.2x-1890.2	0.9996	0.8	2.5
TCBQ	1.0-50.0	y=5593.5x-4729.5	0.9992	0.7	2.0

*y*: peak area; *x*: mass concentration, μg/L.

#### 2.4.2 样品回收率与精密度

通过在草鱼、小龙虾、金鲳鱼空白基质中分别添加其方法定量限1、2、4倍水平的HBQs混合标准溶液进行加标回收试验,每个加标水平设6个平行样,考察方法的准确度和精密度。样品按照1.3节条件提取和净化后,经UPLC-MS/MS检测,结果见表4。不同基质样品中5种HBQs在3个加标水平下的回收率为85.9%~116.5%,相对标准偏差为1.4%~8.2%,实验结果表明建立的方法具有良好的准确度和精密度,可满足分析要求。

**表4:** 5种HBQs在3个加标水平下的回收率和相对标准偏差(*n*=6)

Compound	Spiked level/ (μg/kg)	Grass carp			Cray			Trachinotus ovatus	
		Recovery/%	RSD/%		Recovery/%	RSD/%		Recovery/%	RSD/%
2,5-DCBQ	0.5	104.3	2.6		111.4	2.2		104.8	1.9
	1.0	108.4	2.2		81.9	2.3		85.9	2.5
	2.0	91.8	2.8		105.1	2.5		99.4	2.2
2,6-DCBQ	1.0	95.7	4.8		107.4	3.8		114.1	4.1
	2.0	104.0	4.5		111.4	2.9		107.4	2.6
	4.0	102.2	2.6		109.0	2.7		105.1	2.7
2,6-DBBQ	1.0	97.7	3.4		95.7	1.4		89.6	2.3
	2.0	95.7	2.6		99.5	2.0		101.7	2.9
	4.0	106.8	3.6		91.4	3.0		91.4	3.2
2,6-DCMBQ	2.5	92.0	8.2		109.7	6.7		107.5	6.0
	5.0	103.0	6.9		114.3	2.0		116.5	2.9
	10.0	103.7	3.1		110.7	3.3		111.7	2.7
TCBQ	2.0	90.5	2.4		93.7	0.8		93.8	2.1
	4.0	91.4	2.5		94.6	2.4		95.3	2.1
	8.0	92.5	2.5		95.3	2.1		95.4	0.8

### 2.5 实际样品检测

采用本方法分别对大口黑鲈、鲫鱼、草鱼、金鲳鱼等16个样品进行检测,在1个草鱼样品中检测出2,6-DCMBQ,检测结果低于方法定量限。同时实验结果也说明有必要对水产品中潜在HBQs所带来的健康风险加大关注。

## 3 结论

基于QuEChERS-UPLC-MS/MS建立了同时测定水产品中5种HBQs的分析方法。通过优化仪器条件和前处理方法实现了水产品中5种HBQs的快速测定。方法检出限为0.15~0.8 μg/kg,加标回收率为85.9%~116.5%, RSD小于8.2%。方法前处理技术简单、快速,灵敏度高且稳定性好,可为水产品中HBQs的测定提供可靠的技术支持。
